# Integrating paroxysmal nocturnal hemoglobinuria (PNH) into mainstream hematological curriculum training

**DOI:** 10.1002/hem3.70484

**Published:** 2026-10-05

**Authors:** Morag Griffin, Richard Kelly, Colm Bradley, Sam Salek, Richard Soutar, Argiris Symeonidis

**Affiliations:** ^1^ St James Hospital, Leeds Teaching Hospital NHS Trust Leeds United Kingdom; ^2^ University of Glasgow Glasgow United Kingdom; ^3^ University of Hertfordshire Hatfield United Kingdom; ^4^ Beatson, West of Scotland Cancer Centre Glasgow United Kingdom; ^5^ University of Patras Patras Greece

Paroxysmal nocturnal hemoglobinuria (PNH) is an ultra‐orphan life‐threatening disorder characterized by chronic intravascular hemolysis (IVH) and thrombosis.^1^ Prior to eculizumab approval, patients with PNH had a median survival of between 10 and 22 years^2^, ^3^ with thrombosis accounting for 22%–67% of death in case series and 29%–44% of patients having at least one event.^2^, ^4^ Since the introduction of complement inhibitors, life expectancy in countries where treatment is available has nearly normalized.^5^ As symptoms of PNH can vary and are often non‐specific, patients can present to a variety of medical specialties, leading to delays in diagnosis and appropriate treatment.

Here, we address issues around underdiagnosis and misdiagnosis of PNH and provide an evidence‐based overview of current educational gaps and diagnostic challenges facing hematologists. We describe in more detail what the education, and ultimately the knowledge for clinical practice about PNH, of a trainee in hematology should include. This is an activity of the Gaucher Disease/Rare Disease Task Force, an independent Sub‐Committee of the Granulocytes and Constitutional Marrow Failure Syndromes' Scientific Working Group of EHA, and the description which follows will be submitted for approval by the EHA Curriculum‐Exam Committee.

## BACKGROUND—BIOLOGY AND BIOCHEMISTRY

Hematologists and scientists involved in PNH diagnosis and treatment need to be aware that the mutations which cause PNH are acquired in the PIGA gene, located on the X chromosome, at position p22.2. PIGA codes for glycophosphatidylinositol‐anchored proteins, which include CD55 and CD59, both are negative regulators of complement activation. Men and women are equally affected by PNH, as PIGA mutations are acquired after lyonization. PNH usually presents in adults in their 30s, but can present at any age, including childhood.^6^ The prevalence of PNH with any clone size is 15.9 per million in Europe; this includes patients with small PNH clones, with other hematological conditions.^7^ Published/quoted prevalence data is often lower at 0.5–1.5 cases per million, due to the consideration of clinically significant PNH.

The complement system is part of the innate and adaptive immune response, designed to neutralize and destroy pathogens invading the systemic circulation. CD55 inhibits complement activation at complement protein C3b, and CD59 controls terminal complement activation. In PNH, loss of CD55 and CD59 results in uncontrolled complement activation and subsequent IVH, resulting in free hemoglobin release. Free hemoglobin is a nitric oxide scavenger leading to loss of smooth muscle relaxation and consequently to several PNH symptoms.

Some PNH blood cells have a partial CD55 and CD59 deficiency leading to complement inhibition reduction, whereas other cells have a complete deficiency with complete loss of complement inhibition. PNH cells with a complete deficiency are much more susceptible to hemolysis than cells with a partial deficiency.

## ASPECTS OF DISEASE PATHOPHYSIOLOGY

PNH arises on a background of bone marrow failure, so all patients with aplastic anemia (AA) and hypoplastic myelodysplastic syndrome (MDS) should have a PNH screen.^1^, ^8^

The size of a PNH clone can vary over time. An increase in the proportion of PNH cells is more common if patients are receiving immunosuppressive therapy for bone marrow failure. The proportion of PNH cells requires monitoring every 3 months for the first year after diagnosis, and less frequently (6–12 monthly) thereafter.^8^

Patients with PNH are at increased risk of thrombosis, due to complement activation, release of pro‐inflammatory cytokines and platelet activation. In general, the higher the PNH clone, the higher the risk of a thrombotic event. Before anti‐complement treatment, thrombosis was the highest cause of death accounting for 22%–67% of deaths. The cumulative thrombosis incidence over 8–10 years is between 23% and 30% (pre‐eculizumab era).^2^, ^5^, ^9^

## CLINICAL MANIFESTATIONS AND SPECIFIC DISEASE FEATURES

PNH symptoms mainly arise from IVH, with patients experiencing anemia/fatigue (64.0%–80%), hemoglobinuria (45.5%), breathlessness (23.1%–45%), abdominal pain (18.6%–35%), dysphagia (7.3%–16%), and erectile dysfunction (13.5%–24% male patients).^10^, ^11^

Due to variable symptom presentation, raising disease awareness amongst all healthcare professionals is important. Long diagnostic delays can occur resulting in significant morbidity and a reduced quality of life, as well as unnecessary investigations.

Thrombosis can be the presenting event in PNH. In total, 70%–80% of thromboses are venous, and 20%–30% are arterial. Deep vein thrombosis or pulmonary emboli are common features constituting 20%–33% of events, with intra‐abdominal events in 30%–40% of cases.^10^ All patients with unexplained thrombosis/recurrent thrombosis or cytopenias, even without any manifestation of hemolysis, should be tested for PNH.

## REQUIRED KNOWLEDGE: LABORATORY LEVEL

PNH findings are those of direct antiglobulin test negative hemolytic anemia. Hemoglobin levels may vary and become substantially low, haptoglobin levels are low, and the lactate dehydrogenase (LDH) level is high especially during acute hemolytic episodes. The total and indirect bilirubin may be elevated. The reticulocyte count is usually elevated; however, for patients with concurrent AA, this may be limited. High disease activity markers include an LDH > 1.5 × upper limit normal, high d‐dimers, and anemia.^11^ Blood film changes can be minimal; red cell fragments are NOT in keeping with PNH and other MAHA causes should be sought.

PNH testing should be performed in an accredited laboratory participating in external quality assessment programmes, using high‐sensitivity flow cytometry on peripheral blood, assessing red cells using CD55 and CD59 markers, neutrophils using CD24, CD66b and CD16 (minimum of two reagents) and the monocytes using CD14. FLAER, a bacterial lysin‐based technique, binds specifically to GPI anchor proteins, is considered by current guidelines and the International PNH Interest Group as an accurate, sensitive method for detecting granulocyte and monocyte PNH cells.^12^

It is essential to assess both the white and red cell clone as patients with hemolytic PNH will often have a lower red cell clone, due to IVH or blood transfusions.^12^ Bone marrow examination is not routinely required, unless there are concurrent cytopenias, raising the possibility of bone marrow failure.

Baseline imaging should be considered to assess for occult PNH complications including an echocardiogram to assess right pulmonary artery pressure and an abdominal ultrasound to assess hepatic and splenic blood flow. Disease markers for monitoring to assess response to treatment include serum LDH, hemoglobin, reticulocyte count, renal and liver function tests, and PNH clone.

## DIAGNOSTIC APPROACH—DIFFERENTIAL DIAGNOSIS

Taking into consideration the spectrum of the clinical manifestations and the laboratory findings, it is evident that PNH should be included in the differential diagnosis of diseases associated with DAT‐negative hemolytic anemia, unexplained cytopenias, bone marrow hypoplasia, and unexplained or recurrent thrombosis.^13^ See Table 1 for indications for PNH testing.

**Table 1:** Diagnostic approach—differential diagnosis.

Clinical indications for PNH testing	
Intravascular hemolysis	
Unexplained hemolysis with	Iron deficiency Abdominal pain or cramps Esophageal spasm Cytopenias
Acquired Coombs negative hemolytic anemia	
Thrombosis with unusual features	Unusual sites: hepatic, portal, splenic, splanchnic, cerebral, dermal, pulmonary embolism Accompanied with hemolytic anemia Unexplained cytopenia
Bone marrow failure	Suspected or proven aplastic anemia Myelodysplasia particularly with a hypoplastic marrow Cytopenia of unknown etiology

Abbreviation: PNH, paroxysmal nocturnal hemoglobinuria.

Disease classification of PNH is divided into three categories, see Table 2. PNH is not a “static” disorder, and patients can transition across different disease states during their lifetime.^14^ Clonal evolution may occur in PNH due to underlying bone marrow disorders. If clonal evolution is considered, a bone marrow examination is indicated.

**Table 2:** Disease classification, clinical subtypes, and tips for diagnosis.^14^

Category	Rate of intravascular hemolysis^a^	BM	Flow cytometry
Classic PNH	Florid (markedly abnormal LDH often with episodic macroscopic hemoglobinuria)	Cellular BM with erythroid hyperplasia and normal or near‐normal morphology^b^	Large population (>50%) of GPI‐AP–deficient PMNs^c^
PNH in the setting of BM failure syndrome^d^	Mild (often with minimal abnormalities of biochemical markers of hemolysis)	Evidence of a concomitant BM failure syndromes^d^	Although variable, the percentage of GPI‐AP–deficient PMNs is usually relatively small (<10%)
Subclinical PNH	No clinical or biochemical evidence of intravascular hemolysis	Evidence of concomitant BM failure syndrome^d^	Small (<1%) population of GPI‐AP–deficient PMNs detected by high‐resolution flow cytometry

Abbreviations: BM, bone marrow; GPI‐AP, glycosylphosphatidylinositol‐anchored proteins; LDH, lactate dehydrogenase; PMN, polymorphonuclear cells; PNH, paroxysmal nocturnal hemoglobinuria.

^a^ Based on macroscopic hemoglobinuria, serum LDH concentration, and reticulocyte count.

^b^ Karyotypic abnormalities are uncommon.

^c^ Analysis of PMNs is more informative than analysis of red blood cells (RBCs) due to selective destruction of GPI‐AP–deficient RBCs.

^d^ Aplastic anemia or low‐risk myelodysplastic syndrome (MDS).

## TREATMENT APPROACHES

PNH can present in the context of another bone marrow disorder or de novo. Patients with a clinically significant PNH cell clone may require anti‐complement treatment. Patients with other bone marrow failure disorders should be optimized for their bone marrow failure as per guidelines, for example, AA treated with ATG and ciclosporin or hematopoietic stem cell transplant irrespective of the PNH.^8^

The United Kingdom complement‐inhibitor treatment indications include PNH‐related thrombosis (clinical emergency),^13^ symptomatic IVH with anemia and a raised LDH > 1.5 upper limit normal, PNH complications (renal impairment or pulmonary hypertension), and pregnancy (PNH clone > 20% eculizumab only).

## SERVICE PROVISION AND SPECIALIST CARE

Due to the rarity of PNH, and the probability of patients presenting to other specialties, for example, respiratory with pulmonary emboli, urology with hemoglobinuria misdiagnosed as “hematuria,” we highlight the importance of including PNH in hematology curricula, but also for trainees to gain experience in a center with patients diagnosed and treated for PNH. Treatment is often individualized, and with the development of an increasing number of complement inhibitors, who to commence on treatment and which treatment to commence can be challenging. Often individual clinicians are managing singular patients with PNH. This is challenging for the individual clinicians and patient, who may feel isolated. It is therefore advised that patients are reviewed by a specialist with an interest in PNH, with a shared care approach between the local hematology clinician and the specialist center.

Clinical nurse specialist support is essential for managing patients with PNH, providing patient information, support and liaising with local hospitals when acute issues arise.

Patient support groups are also a good resource to signpost patients to, often offering meetings, webinars and access to psychological support.

## CONCLUSIONS

A PNH curriculum should be included in the main body of education in hematology. Moreover, all hematology departments, which are recognized as training centers for the specialty of hematology, but do not have experience in diagnosing and treating patients with PNH, should allow trainees and young hematologists to visit expert PNH centers for short periods of time, to obtain experience on this disease. In this way, all hematologists will obtain experience with this disease.

## AUTHOR CONTRIBUTIONS

**Morag Griffin:** Conceptualization; writing—original draft; writing—review and editing. **Richard Kelly:** Writing—review and editing. **Colm Bradley:** Conceptualization; writing—review and editing. **Sam Salek:** Writing—review and editing. **Richard Soutar:** Writing—review and editing. **Argiris Symeonidis:** Writing—review and editing.

## CONFLICT OF INTEREST STATEMENT

The authors declare no conflicts of interest.

## FUNDING

This research received no funding.

## Data Availability

The data that support the findings of this study are available on request from the corresponding author. The data are not publicly available due to privacy or ethical restrictions.
